# Global research trends in signaling pathways of spasmolytic polypeptide-expressing metaplasia pathogenesis: a bibliometric analysis

**DOI:** 10.3389/fonc.2025.1597221

**Published:** 2025-12-17

**Authors:** Qiumiao Xu, Zhijiang Dai, Chengjin Peng, Qinghong Yan, Xiaoting Chen, Tongwei Liu, Ran Zhang, Guoxin Huang, Xinyao Liu, Jingbin Wang

**Affiliations:** 1 Department of Spleen and Stomach, Shenzhen Hospital (Futian) of Guangzhou University of Chinese Medicine, Shenzhen, China; 2 The Sixth Clinical Medical College, Guangzhou University of Chinese Medicine, Shenzhen, China

**Keywords:** bibliometrics, CiteSpace, knowledge mapping, signaling pathway, spasmolytic polypeptide-expressing metaplasia, VOSviewer

## Abstract

**Background:**

Signaling pathways associated with spasmolytic polypeptide-expressing metaplasia (SPEM) pathogenesis play a critical role in disease development, particularly in gastric cancer precursor lesions. However, global research trends in signaling pathways in SPEM pathogenesis remain undetermined. This study aims to fill this gap by conducting a comprehensive bibliometric analysis to map the field, identify key insights, and guide future research directions.

**Methods:**

Articles and reviews were retrieved from the Web of Science Core Collection up to June 8 2025. Bibliometric analysis and knowledge mapping were conducted via CiteSpace and VOSviewer.

**Results:**

A total of 89 papers from 221 institutions, 719 authors, and 56 journals across 21 countries/regions were included. The number of publications is growing slowly. *Gastroenterology* led in publication and co-citation counts. The United States and China were at the top in terms of publication numbers. Goldenring JR emerged as the most co-cited and most published author. Key findings indicate limited collaboration despite a foundation of ten pivotal articles. Research elucidating gene-pathway interplay and cytokine roles in SPEM pathogenesis has consequently sharpened the focus on developing pharmacological agents that target these pathways, defining a pivotal new direction for combating gastric precancerous lesions.

**Conclusion:**

By systematically reviewing signaling pathways in SPEM pathogenesis, this study provides critical guidance for future research strategic planning and collaboration. It highlights the urgent need for strengthened interdisciplinary and global partnerships to drive progress.

## 1 Introduction

Gastric cancer (GC) is one of the most common malignant tumors in the world, with the fifth highest incidence and fifth highest mortality rates of all cancers, respectively (1). In China, the disease burden of GC is particularly prominent, with the third highest incidence and mortality rates among all cancer types (2). *Helicobacter pylori* (*H. pylori*), classified as a Group I carcinogen by International Agency for Research on Cancer (IARC), colonizes the gastric mucosa (3, 4). The cytotoxin-associated gene A positive (CagA+) *H. pylori* strain stimulates parietal cells to secrete Sonic hedgehog (SHh), which upregulates Programmed Cell Death Ligand 1 (PD-L1) expression on spasmolytic polypeptide-expressing metaplasia (SPEM) cells (5, 6). The expression of PD-L1 by SPEM cells, identified here as a key survival mechanism during chronic inflammation, contributes to the persistence of infection and subsequent progression to gastric cancer (7). Epidemiological studies indicate that approximately 89% of noncardial gastric cancer (NCGC) are strongly associated with chronic *H. pylori* infection (8). During the process of gastric carcinogenesis, the gastric mucosa typically undergoes a multistage evolutionary cascade known as the Correa Cascade (9, 10). This cascade involves a sequence of events that include chronic inflammation, atrophic gastritis, intestinal metaplasia (IM), and dysplasia, ultimately resulting in adenocarcinoma. However, the molecular mechanisms underlying the key precancerous lesions in this cascade have not been fully elucidated, and in particular, significant knowledge gaps remain in the mechanisms driving SPEM, which has received much attention in recent years.

SPEM is a reversible precancerous lesion of the stomach, defined as metaplastic foci arising from aberrant proliferation of mucous neck cells and isthmus stem cells after injury to the corpus glands. The functional consequence of this cellular reprogramming is the replacement of the acid-secreting parietal and digestive enzyme-producing chief cells with Trefoil Factor 2 (TFF2) - and Mucin 6 (MUC6) -expressing mucinous cells, thereby creating a key transitional state in gastric carcinogenesis (11). Histologically, it is characterized by the replacement of parietal cells with mucin-rich, columnar cells expressing TFF2, MUC6 and CD44 variant isoform 9 (CD44v9), converting the glandular architecture from an acid-secreting to a mucin-secreting phenotype (12–16). In experimental murine models of acute parietal cell loss, SPEM emerges as a rapid and reversible repair response, often detectable within 72 hours after glandular injury and subsiding once repair is complete (17–20). However, in the clinical context of chronic human pathologies such as *H. pylori* infection, SPEM presents as a persistent metaplastic lesion. This physiological repair program appears to be co-opted and sustained by chronic inflammatory stimuli, leading to a stable, precancerous state that is slow to regress even after the removal of the initial insult (21). This process relies on TFF2-mediated activation of the MAPK/ERK and PI3K/Akt signaling pathways to achieve mucosal barrier reconstruction by promoting cell migration and inhibiting apoptosis (22, 23). However, under the stimulation of persistent damage from chronic Helicobacter pylori infection or dietary insults, SPEM may overcome homeostatic regulation through the dual mechanisms of oxyntic atrophy and chronic inflammation. The loss of parietal cells disrupts essential signaling pathways including TGF-α and hedgehog signaling, while sustained inflammation promotes transcriptomic reprogramming of chief cells through factors such as MIST1 downregulation. This metaplastic progression can evolve into irreversible intestinal metaplasia and ultimately dysplasia, driving gastric carcinogenesis through these well-established molecular mechanisms described in previous studies (11, 24).

Notably, TFF2’s critical role in SPEM development exemplifies this functional specialization within the trefoil factor family. As the molecular hallmark of SPEM, TFF2 not only mediates acute mucosal repair through MUC6 co-expression and signaling pathway activation, but also demonstrates the delicate balance between physiological regeneration and pathological progression. This duality mirrors the broader functional dichotomy observed across the trefoil factor family - where TFF2’s gastric-specific protection collaborates with TFF1’s epithelial adhesion regulation and TFF3’s intestinal restitution mechanisms to maintain gastrointestinal homeostasis (25, 26).

Recent studies have revealed a correlation between the malignant transformation of SPEM and the aberrant activation of multiple signaling pathways (27). For example, *H. pylori* activates the Wnt/β-catenin pathway via the CagA virulence factor, which results in β-catenin nuclear translocation and the proliferation of chemotaxis cells (28). Conversely, Telomerase Reverse Transcriptase (TERT) gene deletion significantly inhibits Wnt signaling and alleviates the pathological process of *H. pylori*-induced SPEM (29). *H. pylori* affects cellular behavior through metabolic reprogramming and autophagy pathways, in which Myelocytomatosis viral oncogene homolog (c-Myc) and TFF2 may play key roles (30, 31). Sustained activation of the nuclear factor kappa-B (NF-κB) and signal transducer and activator of transcription 3 (STAT3) pathways in the inflammatory microenvironment creates a self-amplifying, pro-inflammatory circuit. This circuit is driven by the NF-κB/STAT3-induced secretion of cytokines such as interleukin-6 (IL-6) and tumor necrosis factor-alpha (TNF-α), which in turn act as signaling ligands to further activate both pathways, perpetuating a feed-forward loop that exacerbates inflammation and contributes to disease pathogenesis (32). However, the molecular mechanisms underlying the transition of SPEM from reversible repair to irreversible precancer remain to be elucidated.

TFF2 and other biomarkers exhibit dual roles in gastric carcinogenesis (33), reflecting the complex molecular transitions at the “point of no return” (34). Beyond its fundamental, homeostatic role in mucosal defense under physiological conditions, TFF2 exerts protective effects and promotes restitution during acute mucosal injury, its sustained expression under conditions of chronic damage (e.g., persistent H. pylori infection) may contribute to the maintenance and progression of metaplastic lineages, as observed in SPEM (35, 36). When inflammation becomes chronic, however, persistent TFF2 signaling may contribute to carcinogenesis by promoting intestinal metaplasia and angiogenesis through Caudal Type Homeobox 2 (CDX2) activation (37). This functional switch is accompanied by dynamic changes in key molecules: CDX2 transforms from a physiological differentiation factor to an oncogenic driver (38), and p53 mutations progressively accumulate (39). These alterations collectively drive the progression toward a “point of no return”—a critical transition from incomplete intestinal metaplasia to low-grade dysplasia beyond which malignant progression becomes highly likely. Unfortunately, reliable indicators to predict which lesions will cross this threshold remain lacking.

A comprehensive understanding of the pathological mechanisms of SPEM is a core issue that current researchers urgently need to address. Therefore, targeting the relevant regulatory molecules and signaling pathways of SPEM would be a potentially feasible approach to treat SPEM and prevent gastric carcinogenesis. This study aimed to reveal the mechanism of SPEM in gastric precancerous lesions by systematically analyzing research progress on signaling pathways in SPEM pathogenesis, and to provide a theoretical basis for future therapeutic strategies.

To further understand the impact of signaling pathways in the pathogenesis of SPEM, this study uses two commonly used bibliometric tools, CiteSpace (40) and VOSviewer (41), to objectively describe the knowledge areas and emerging trends in signaling pathways in SPEM pathogenesis research in the following four ways. (1) Quantifying and identifying general information in signaling pathways in SPEM pathogenesis research, such as individual impacts and collaborations, by analyzing annual publications, journals, co-cited journals, countries/regions, institutions, authors and co-cited authors. (2) Finding and analyzing the most cited papers by co-identifying cited references to assess the knowledge base of signaling pathways in SPEM pathogenesis. (3) Most importantly, keyword analysis and co-cited reference burst analysis were used to discover the knowledge structure and hotspot evolution to further identify (42, 43) what is being researched in the field of signaling pathways in SPEM pathogenesis and possible future directions.

## 2 Materials and methods

### 2.1 Database and search strategy

**Figure 1:**
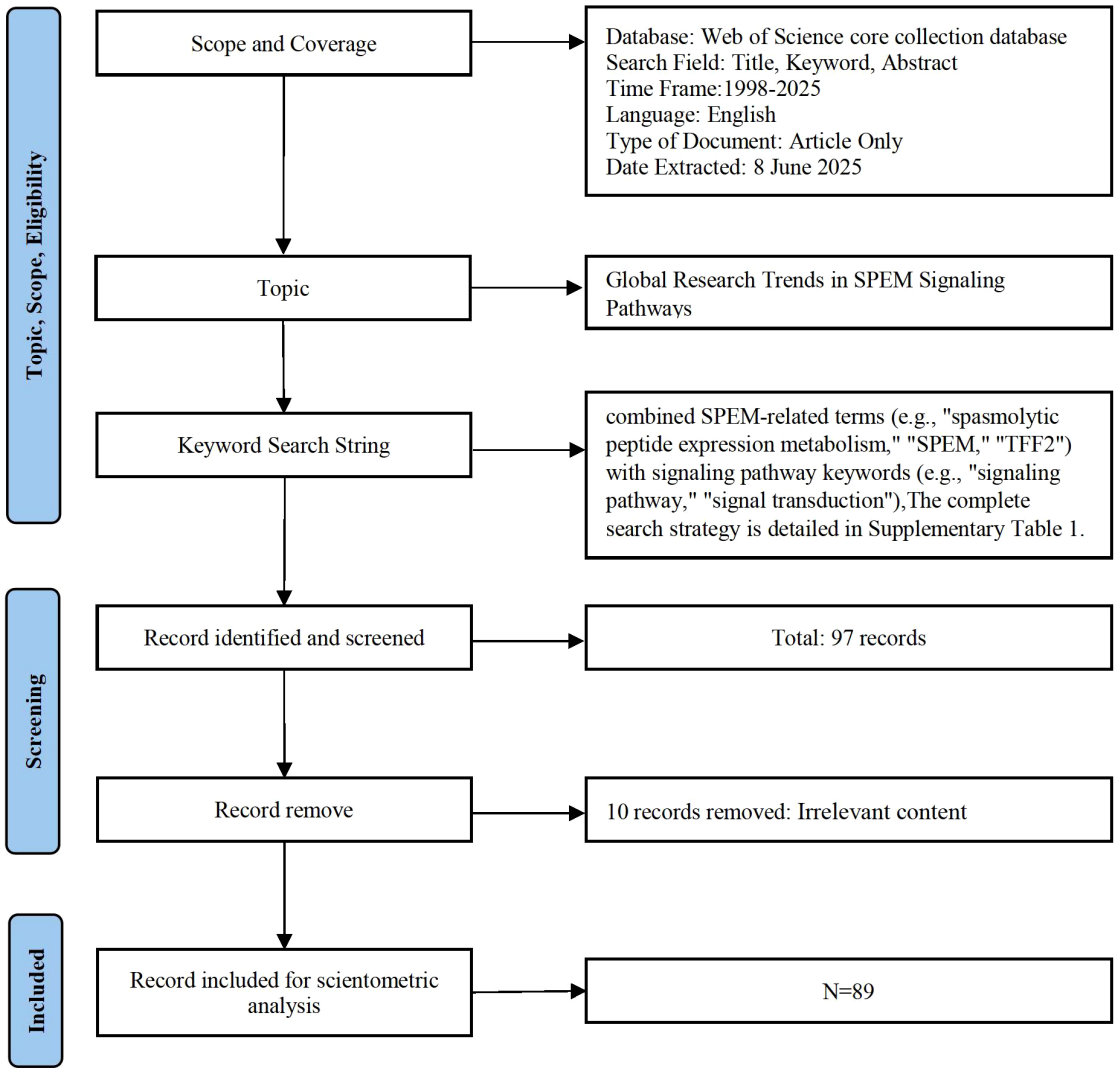
The research flow chart.

This study employed bibliometric analysis to investigate signaling pathways in SPEM pathogenesis in the Web of Science Core Collection (WoSCC) from its inception until June 8, 2025. The search combined SPEM-related terms (e.g., “spasmolytic peptide expression metabolism,” “SPEM,” “TFF2”) with signaling pathway keywords (e.g., “signaling pathway,” “signal transduction”) using the Boolean operator “AND.” Only English-language articles were included, while non-research publications (e.g., reviews, editorials, conference abstracts, letters) were excluded. The complete search strategy is detailed in **Supplementary Table 1**. Two researchers independently screened 97 publications, excluded 8 irrelevant records, and ultimately included 89 articles for analysis (**Figure 1**). The data were exported from WoSCC and normalized to ensure consistency.

### 2.2 Data analysis and visualization

The data were processed via Microsoft Excel 2016 for database management and annual publication analysis, and the journal metrics were sourced from the Web of Science Journal Citation Reports. Bibliometric analyses were conducted via VOSviewer 1.6.20 and CiteSpace 6.4.R1. Data were cleaned to standardize terms (e.g., unifying “human spasmolytic polypeptide” and “polypeptide-expressing metaplasia” to “SPEM”) and abbreviations. VOSviewer creates network, density, and overlay maps to visualize relationships among authors, institutions, and keywords. Fractional counting was applied to assess collaboration strength, with a maximum of 25 authors per document. In CiteSpace, centrality (a metric ranging from 0 to 1) was used to identify pivotal nodes (e.g., publications, keywords, or authors) that function as bridges between research clusters, with values ≥0.1 indicating high interdisciplinary or conceptual significance. The CiteSpace settings included a time span of 1998 - 2025, year slices of 1, data cropping via the minimum spanning tree algorithm and path simplification network, and selection criteria of G value = 25. This approach enabled a detailed evaluation of research trends, key collaborations, and influential publications in the field of signaling pathways in SPEM pathogenesis.

## 3 Results

### 3.1 Annual growth trend and annual number of citations

**Figure 2:**
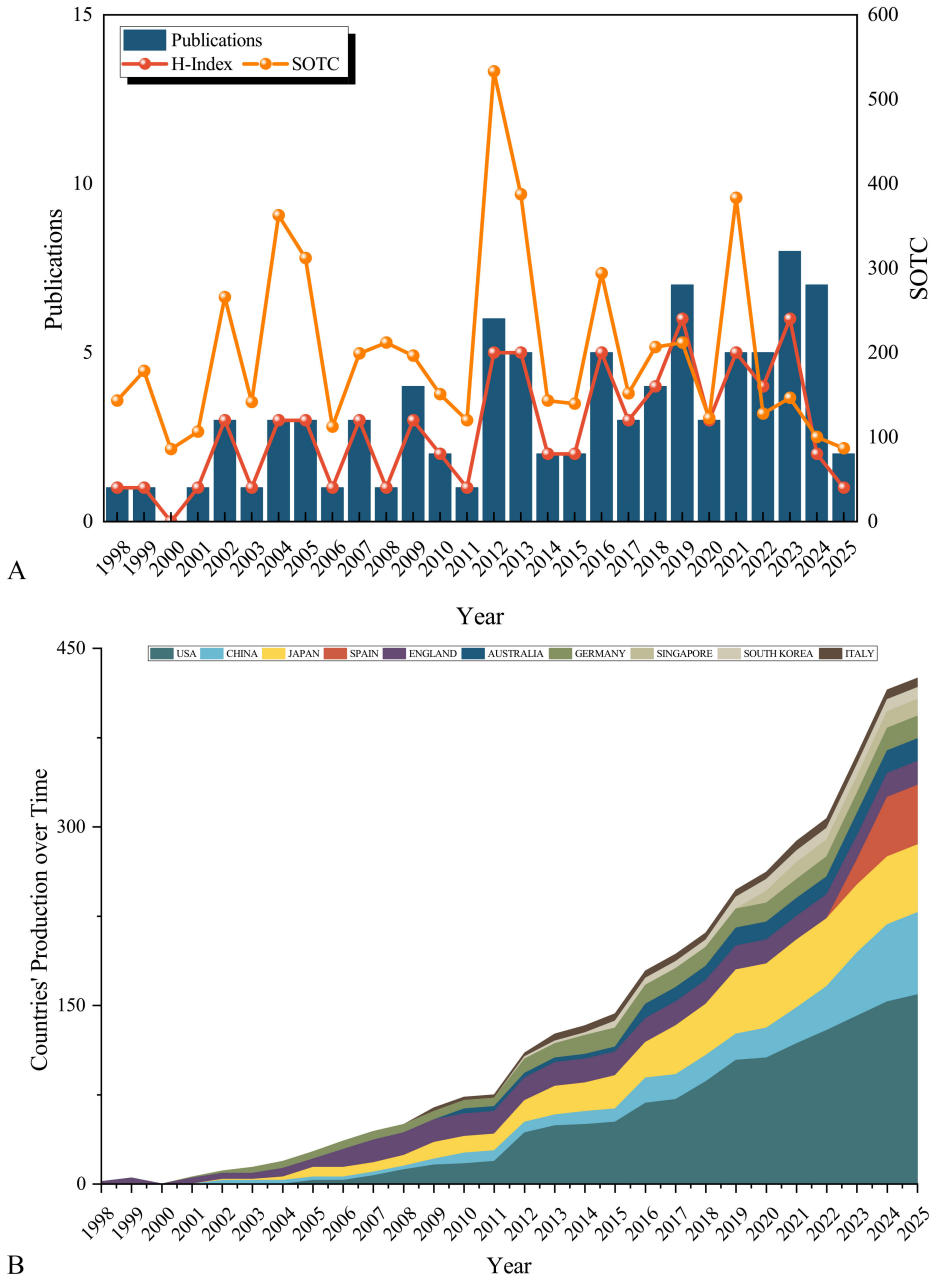
Annual growth trend and citation analysis on signaling pathways in SPEM pathogenesis. **(A)** Annual publication volume on signaling pathways in SPEM pathogenesis; SOTC: Sum of the Times Cited; **(B)** Composition ratio of articles published in top 10 countries.

Annual publications increased steadily from 1-3 (1998-2011) to a peak of 8 in 2023 (**Figure 2A**). The H-index (a measure of both productivity and citation impact, where a value of 6 means 6 publications each cited at least 6 times) showed similar growth, reaching 6 in 2023. Total citations (sum of the times cited, SOTC) peaked in 2004 (323), 2012 (522), and 2021 (347). Recent years (2023-2024) exhibited higher output but lower citations, consistent with expected citation lags. Notably, 2012 showed concurrent peaks in publications (6), H-index (5), and citations (522). Country-level analysis (**Figure 2B**) demonstrated the USA, China, and Japan as dominant contributors. China exhibited particularly rapid growth. The UK maintained consistent output, while Spain showed remarkable recent growth.

### 3.2 Journals and co-cited journals

**Figure 3:**
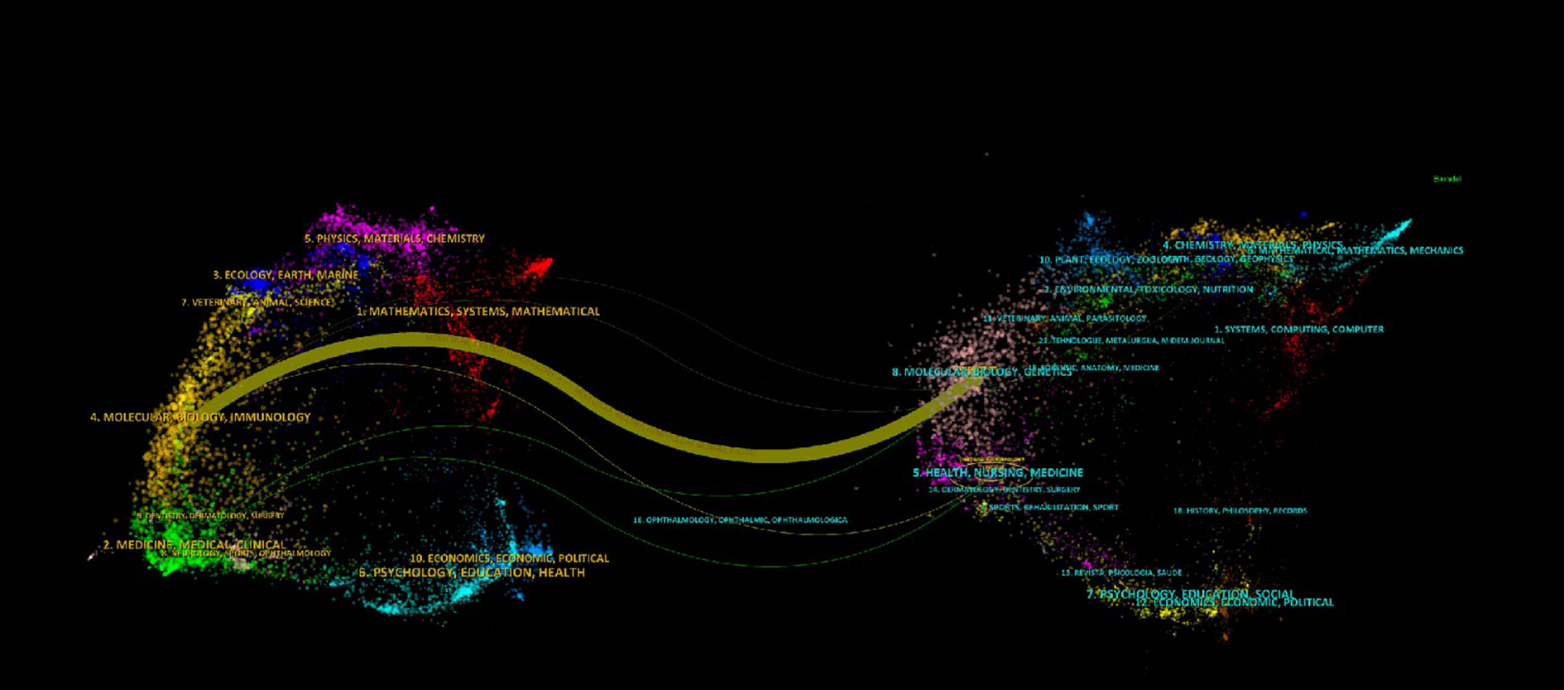
Dual-map overlay of journals shows knowledge flows (colored curves) between citing (left) and cited (right) journals, with node size indicating influence and colors representing disciplines. Thicker curves denote stronger citation relationships.

Our analysis of 89 publications from 56 journals revealed a distinct core-periphery distribution in research output. Notably, Gastroenterology emerged as the leading journal, contributing the highest number of articles (n=9, 10.11%) and maintaining the highest impact factor among the surveyed publications. This dominance underscores its pivotal role in disseminating influential research within the field. High-impact journals like Gut (IF = 23.1) and Cancer Research (IF = 12.5) publish fewer but highly cited articles, driving breakthroughs (**Tables 1**, **2**). Knowledge graph analyses reveal multidisciplinary clusters (e.g., medicine-math, ecology-earth sciences), with Gastroenterology and Gut frequently cited, reflecting SPEM research’s integration with molecular biology and genomics. The dual-map overlay technique identifies influential journals in the field by analyzing citation relationships between source publications and their referenced works. Biplot analysis highlights molecular biology’s internal circulation, with journals such as Gastroenterology, Gut and Cancer Research being the main sources of knowledge (**Figure 3**). Future efforts should leverage interdisciplinary potentials by publishing in high-impact cross-field journals to bridge basic-clinical gaps and maximize knowledge value.

**Table 1:** Top 10 journals in the field on signaling pathways in SPEM pathogenesis in terms of number of publications.

Journal	Publications	Percent (%)	IF (2024)	JCR division	Country
GASTROENTEROLOGY	9	10.11%	25.10	Q1	USA
AMERICAN JOURNAL OF PHYSIOLOGY-GASTROINTESTINAL AND LIVER PHYSIOLOGY	6	6.74%	3.30	Q1	USA
CELLULAR AND MOLECULAR GASTROENTEROLOGY AND HEPATOLOGY	4	4.49%	7.40	Q1	USA
GUT	4	4.49%	25.80	Q1	ENGLAND
INTERNATIONAL JOURNAL OF MOLECULAR SCIENCES	4	4.49%	4.90	Q1	SWITZERLAND
JOURNAL OF PATHOLOGY	4	4.49%	5.20	Q1	ENGLAND
HUMAN PATHOLOGY	3	3.37%	2.60	Q2	USA
CANCER RESEARCH	2	2.25%	16.60	Q1	USA
INFECTION AND IMMUNITY	2	2.25%	2.80	Q3	USA
JOURNAL OF BIOLOGICAL CHEMISTRY	2	2.25%	3.90	Q2	USA

**Table 2:** Top 10 journals in the field on signaling pathways in SPEM pathogenesis by citations.

Journal	Publications	IF (2024)	JCR Division	Country
GASTROENTEROLOGY	468	25.10	Q1	USA
GUT	170	25.80	Q1	ENGLAND
CANCER RESEARCH	119	16.60	Q1	USA
JOURNAL OF BIOLOGICAL CHEMISTRY	99	3.90	Q2	USA
NATURE	99	48.50	Q1	ENGLAND
PROCEEDINGS OF THE NATIONAL ACADEMY OF SCIENCES OF THE UNITED STATES OF AMERICA	96	9.10	Q1	USA
JOURNAL OF PATHOLOGY	82	5.20	Q1	ENGLAND
CELL	74	42.50	Q1	USA
SCIENCE	73	45.80	Q1	USA
JOURNAL OF CLINICAL INVESTIGATION	72	13.60	Q1	USA

### 3.3 Country/region and institution

**Figure 4:**
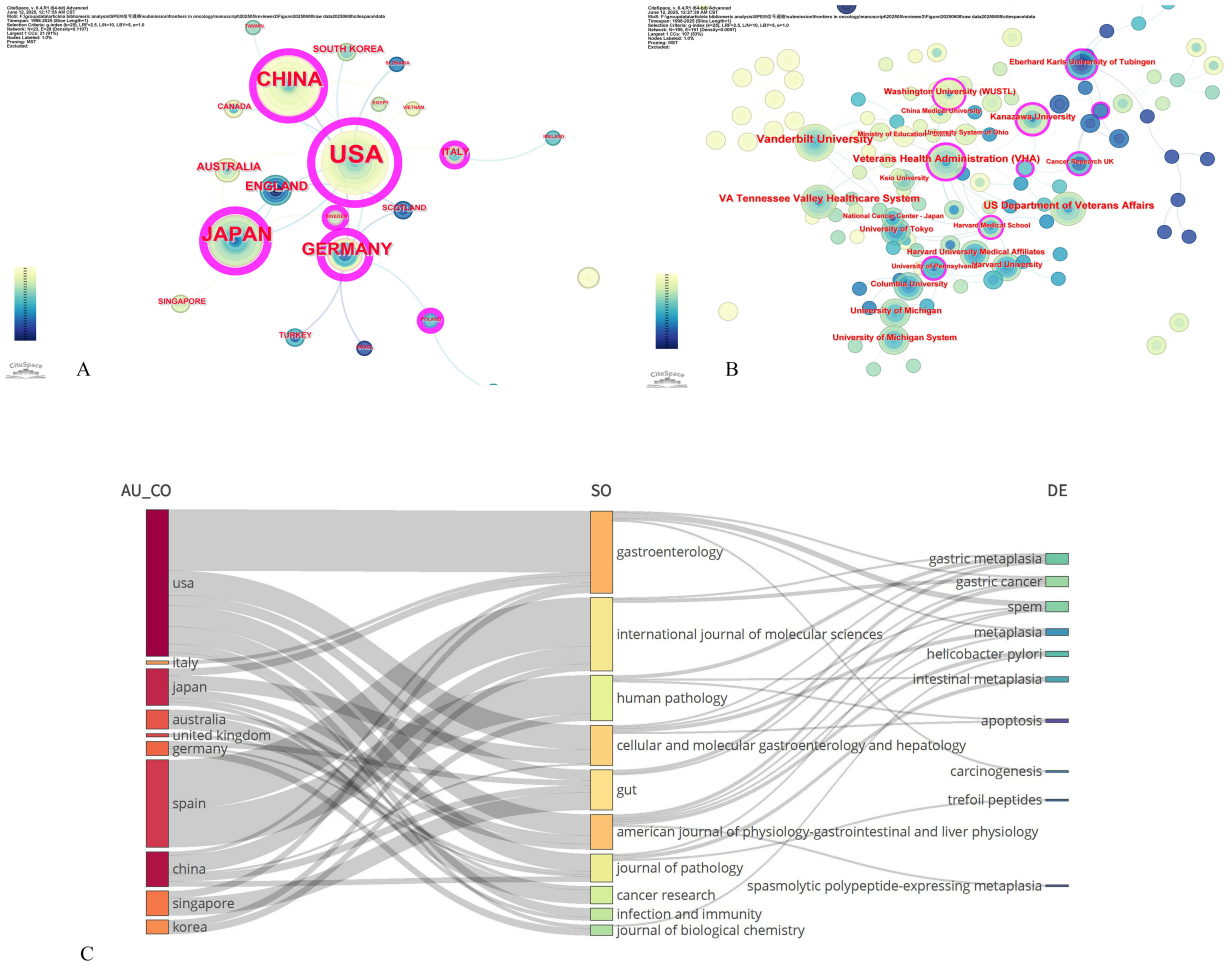
Contributions of countries and institution to signaling pathways in SPEM pathogenesis. **(A)** Country co-occurrence map; **(B)** Institution co-occurrence map. Note: size response frequency, connections represent co-occurring relationships, chronological colors represent years, from grey to red for the span of years from 1998 to 2025, and purple circles on the outer rim of the nodes represent high mediator centrality (>0.1). **(C)** The Sankey diagram illustrates the interrelationships among article characteristics, including authors, journals, and keywords, with features arranged from top to bottom in descending order of frequency.

The multinational collaboration network included 221 institutions across 21 countries, yielding 89 publications. Productivity analysis revealed marked geographic disparities: the United States led with 41 publications (33.06%), focusing on TFF2-mediated mucosal repair and neoplastic transformation in SPEM pathogenesis (44–46). China follows with 20 studies (16.13%), emphasizing traditional medicine interventions (47) and clinicopathological correlations (48). Japan contributes 17 publications (13.71%), specializing in *H. pylori* pathogenesis (49). Germany (10, 8.06%) contributed seminal works on mucosal repair–pathology transitions (50, 51) (**Table 3**). CiteSpace analysis identified the U.S., China, Germany, Japan, the U.K., and Italy as pivotal ‘bridge nations’ (median centrality ≥0.10; **Figure 4A**). Literature with high intermediary centrality typically serves as a key link between two fields and is referred to as a “turning point” in CiteSpace, potentially leading to transformative discoveries and acting as a bridge. Despite this, the collaboration network exhibited a sparse core-periphery structure (density=0.1107 after pruning), with intensive cooperation concentrated among the U.S., Germany, Japan, and China, whereas low-income regions remained underrepresented. Institutional analysis (**Figure 4B**) highlighted academic “silos”: 60% of the top-active institutions were U.S., yet cross-border collaborations accounted for only 18% of the output, often constrained by short-term funding mechanisms. This fragmented landscape underscores the untapped potential for leveraging geographic strengths through structured multinational consortia.

**Table 3:** Top 10 countries and organizations with publications on signaling pathways in SPEM pathogenesis.

Region	Publications	Percent (%)	Centrality	Institution	Publications	Percent (%)	Country
USA	41	33.06%	0.97	Vanderbilt University	10	3.27%	USA
CHINA	20	16.13%	0.23	Veterans Health Administration	7	2.29%	USA
JAPAN	17	13.71%	0.50	US Department of Veterans Affairs	7	2.29%	USA
GERMANY	10	8.06%	0.73	VA Tennessee Valley Healthcare System	7	2.29%	USA
ENGLAND	5	4.03%	0.13	Kanazawa University	5	1.63%	JAPAN
AUSTRALIA	4	3.23%	0.00	Washington University	5	1.63%	USA
ITALY	3	2.42%	0.16	University of Michigan	5	1.63%	USA
SOUTH KOREA	3	2.42%	0.00	University of Michigan System	5	1.63%	USA
SPAIN	3	2.42%	0.00	Eberhard Karls University of Tubingen	4	1.31%	GERMANY
CANADA	2	1.61%	0.00	Harvard University	4	1.31%	USA

**Figure 4C** presents a country-specific heatmap of research priorities in signaling pathways underlying SPEM pathogenesis, visually complementing our global bibliometric trends analysis. Integrated VOSviewer/CiteSpace analysis generated radar charts that quantify research emphasis across five thematic axes: (1) TFF2/SPEM molecular biology, (2) Helicobacter pylori pathogenesis mechanisms, (3) metaplasia-dysplasia-carcinoma progression, (4) mucosal repair/regeneration pathways, and (5) therapeutic targeting and apoptosis regulation.

The visualization reveals distinct national research fingerprints: The United States dominates axes 1 and 3, reflecting its pioneering work in TFF2-mediated neoplastic transformation models. China’s pronounced axis 5 profile aligns with its unique focus on phytochemical modulation of apoptotic pathways in SPEM. Japan’s axis 2 prominence confirms its continued leadership in *H. pylori*-related premalignant lesion studies, while Germany’s axis 4 specialization highlights its expertise in mucosal wound healing responses.

Notably, secondary research hubs (Australia, U.K., Italy, Singapore) exhibit concentrated but impactful contributions - particularly in emerging areas like single-cell transcriptomic mapping of SPEM (Australia) and microbiome-metaplectic niche interactions (Singapore) - forming critical interdisciplinary bridges in our CiteSpace-derived collaboration network. This core-periphery structure, quantitatively identified through betweenness centrality metrics, underscores the evolving global research ecosystem in SPEM pathophysiology.

### 3.4 Authors and co-cited authors

**Figure 5:**
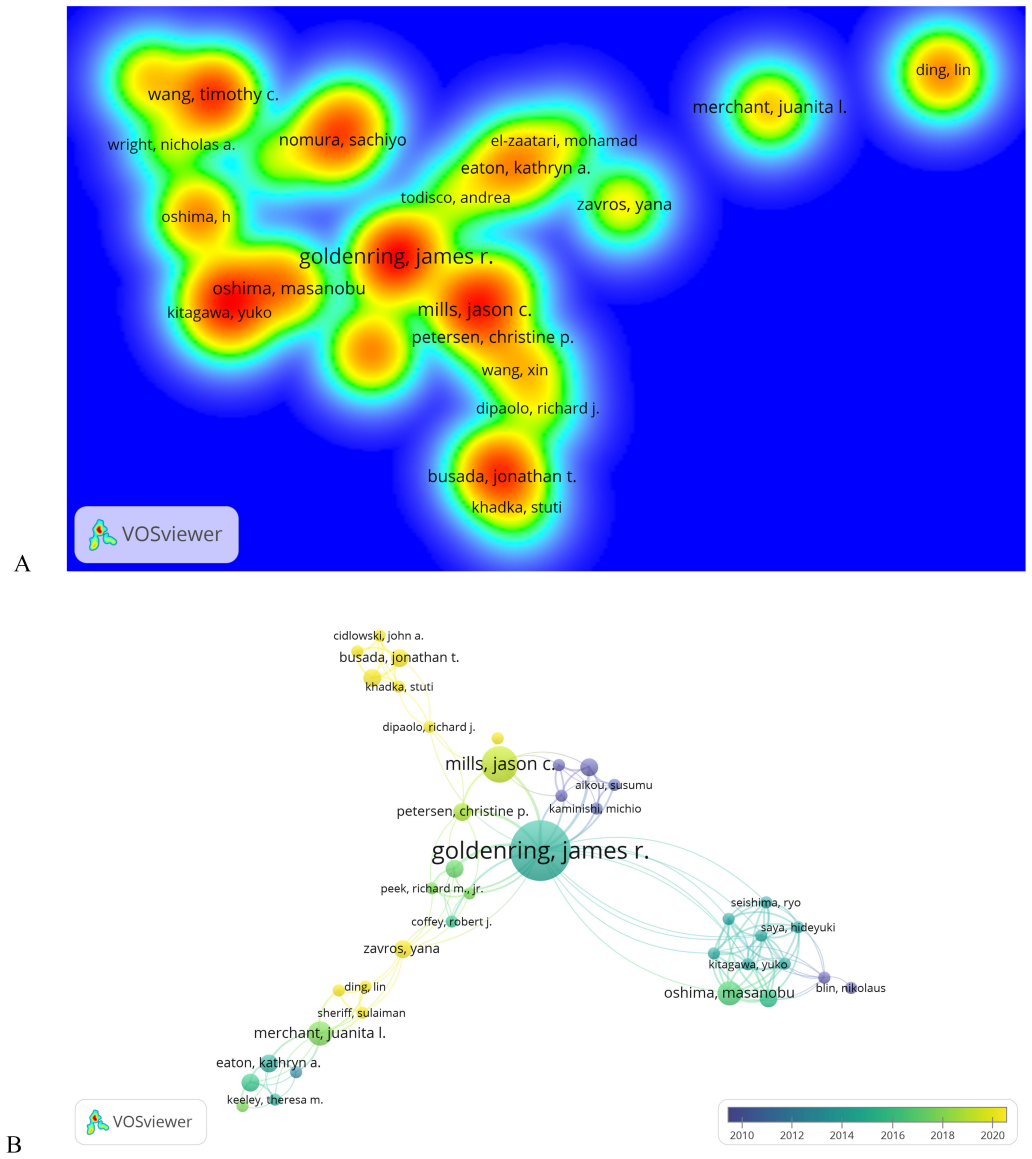
Analysis of authors on signaling pathways in SPEM pathogenesis. **(A)** Author density map on signaling pathways in SPEM pathogenesis domains (T≥2). Note: The size of the label text, the size of the circle, and the area of the yellow area are all positively correlated with the frequency of published literature; **(B)** Co-occurrence plot of co-cited authors on signaling pathways in SPEM pathogenesis domains (T≥5). Note: Connected lines represent authors who see co-occurrence relationships, and nodes of the same color represent the same cluster.

A total of 719 authors have contributed to research on signaling pathways involved in the development and regulation of SPEM, with 85 authors publishing two or more articles. GOLDENRING JR leads with the highest number of publications (n = 10), followed by MILLS JC and OSHIMA M (n = 6 each), and OSHIMA H (n = 5) (**Table 4**). To analyze collaboration patterns, we constructed an author network using the 65 authors who published at least two papers (T ≥ 20) (**Figure 5A**). This knowledge graph highlights partnerships among high-frequency authors. The largest and most prominently colored group in **Figure 5A** consists of GOLDENRING JR and MILLS JC, underscoring their significant contributions to understanding the signaling pathways involved in SPEM pathogenesis. The second-largest group includes OSHIMA M and BUSADA JT.

**Table 4:** Top 10 authors and co-cited authors in the field on signaling pathways in SPEM pathogenesis.

Authors	Publications	Country	H-index	Co-cited author	Citations	Country	H-index
GOLDENRING JR	10	USA	75	GOLDENRING JR	53	USA	75
MILLS JC	6	USA	51	CORREA P	46	USA	83
OSHIMA M	6	JAPAN	61	NAM KT	27	KOREA	43
OSHIMA H	5	JAPAN	22	PETERSEN CP	26	USA	16
MERCHANT JL	4	USA	46	FOX JG	25	USA	101
WANG TC	4	USA	91	KARAM SM	25	UNITED ARAB EMIRATES	30
BLIN N	3	GERMANY	30	NOMURA S	25	JAPAN	20
BUSADA JT	3	USA	13	KATOH M	24	JAPAN	39
CHOI E	3	USA	22	WEIS VG	23	USA	16
DUBEYKOVSKAYA Z	3	USA	3	THIM L	20	DENMARK	59

Co-citation analysis of 3,430 authors (**Figure 5B**) further revealed three thematic domains among 75 influential scholars (citation frequency ≥5): (1) the Hormonal Regulation Group (Cidlowski JA, Busada JT) focused on glucocorticoid signaling in mucosal differentiation, (2) the Metaplastic Transition Group (Mills JC, Petersen CP) investigating SPEM lineage mechanisms, and (3) the Microenvironment Group (Goldenring JR, Peek RM) studying *H. pylori* and stromal interactions, with Goldenring JR emerging as the most interconnected researcher bridging multiple groups.

### 3.5 Keyword co-occurrence, clustering and evolution

**Figure 6:**
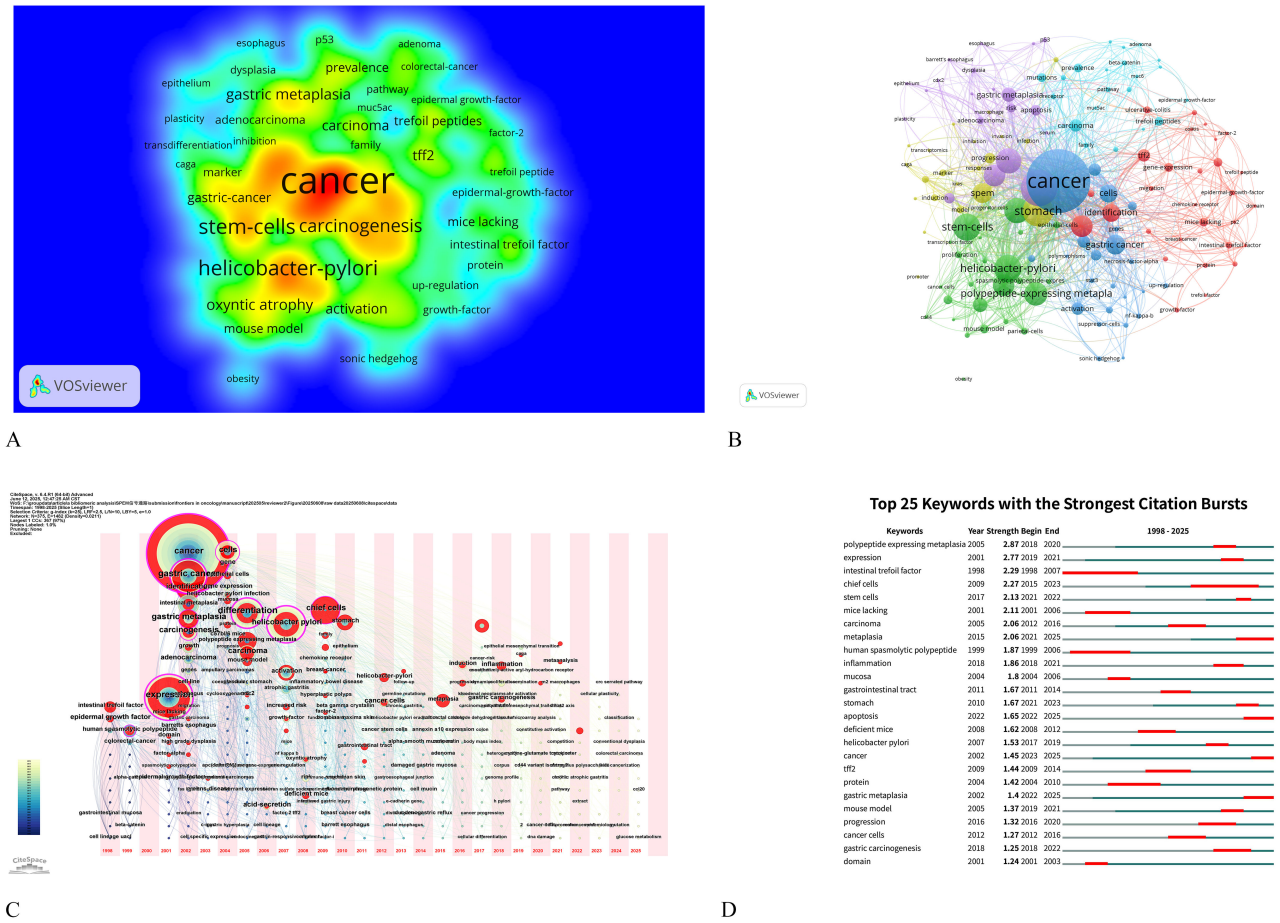
Keyword analysis on signaling pathways in SPEM pathogenesis. **(A)** Density map of terms (T≥2). Note: The size of the label text, the size of the circles, and the area of the yellow region are all positively correlated with the frequency of term occurrence; **(B)** Term co-occurrence graph (T≥5, containing 363 terms, 5 clusters and 178 lines). Note: The size of the node and label text represents the frequency of co-occurrence, the connecting line represents the term see co-occurrence relationship, and the same color node represents the same cluster; **(C)** Map of keyword time zones. Note: The size of the crosses and label text reflects the co-occurrence frequency, and the connecting lines indicate co-occurrence relationships. The colors of the nodes and connecting lines represent different years; **(D)** Burst Keywords Visualization presents the temporal dynamics of high-frequency terms identified through citation burst analysis, where keywords are ranked by their intensity and duration of citation surges, with color-coded bars representing the magnitude of each burst event over specific time periods.

Keywords serve as a synthesis of research themes, where high-frequency terms reflect hotspots and high-centrality terms denote pivotal conceptual bridges. The density map analysis of 629 keywords (**Figure 6A**) revealed “cancer” as the dominant term (n=35, 5.56%), followed by “Helicobacter-pylori” (n=15, 2.38%), “stem-cells” (n=15, 2.38%), “differentiation” (n=14, 2.23%), and “metaplasia” (n=14, 2.23%), highlighting the field’s strong focus on SPEM’s role in gastric carcinogenesis, particularly through *H. pylori*-driven chronic inflammation and cellular reprogramming. The prominence of “stem-cells” and “differentiation” underscores the importance of cellular plasticity in SPEM development, while the high frequency of “metaplasia” reflects its critical position as a precancerous transition in the Correa cascade. This keyword distribution suggests that current research prioritizes understanding the molecular mechanisms linking SPEM to gastric cancer, with an emphasis on infection-related pathways, stem cell dynamics, and metaplastic progression, while also indicating potential gaps in translational applications such as biomarkers and targeted therapies (**Table 5**). Centrality analysis reveals “expression” (0.61) and “Helicobacter pylori” (0.4) as core hubs in SPEM research, framing two key dimensions: molecular mechanisms of gene regulation (particularly TFF2 and MIST1 pathways) and *H. pylori*-driven pathogenesis. These nodes interconnect multiple research clusters, reflecting the field’s dual focus on transcriptional control of metaplasia and infection-mediated carcinogenesis in gastric mucosa. The high centrality of “expression” marks the shift from histological characterization to mechanistic studies of cellular reprogramming, while maintaining *H. pylori* as the predominant etiological factor in SPEM development (**Table 6**).

**Table 5:** Top 20 high-frequency keywords on signaling pathways in SPEM pathogenesis domains.

Rank	Keyword	Cluster	Occurrences	Percent (%)	Rank	Keyword	Cluster	Occurrences	Percent (%)
1	cancer	5	35	5.56%	11	intestinal metaplasia	3	12	1.91%
2	expression	1	20	3.18%	12	gastric cancer	1	11	1.75%
3	stomach	4	17	2.70%	13	spam	2	11	1.75%
4	helicobacter-pylori	2	15	2.38%	14	cells	4	10	1.59%
5	stem-cells	2	15	2.38%	15	identification	1	10	1.59%
6	differentiation	4	14	2.23%	16	gastric metaplasia	5	8	1.27%
7	metaplasia	2	14	2.23%	17	oxyntic atrophy	2	8	1.27%
8	polypeptide-expressing metaplasia	2	13	2.07%	18	activation	1	7	1.11%
9	carcinogenesis	3	12	1.91%	19	carcinoma	3	7	1.11%
10	chief cells	2	12	1.91%	20	inflammation	3	7	1.11%

**Table 6:** Top 15 keywords used in the study for signaling pathways in SPEM pathogenesis in terms of frequency and centrality.

Rank	Keywords	Count	Keywords	Centrality
1	cancer	35	expression	0.61
2	expression	20	helicobacter pylori	0.4
3	helicobacter pylori	16	differentiation	0.27
4	gastric cancer	15	gastric cancer	0.23
5	differentiation	13	human spasmolytic polypeptide	0.19
6	chief cells	12	gastric metaplasia	0.17
7	identification	10	cancer	0.15
8	cells	10	identification	0.15
9	intestinal metaplasia	9	carcinoma	0.14
10	gastric metaplasia	8	chief cells	0.13
11	polypeptide expressing metaplasia	8	carcinogenesis	0.13
12	carcinogenesis	7	gene	0.12
13	carcinoma	7	intestinal trefoil factor	0.12
14	stomach	7	helicobacter-pylori	0.11
15	activation	7	stomach	0.1

The co-occurrence network was resolved into five thematic clusters, each representing distinct research dimensions (**Figure 6B**, **Table 5**). Cluster 1 (red), dominated by “expression” and “identification,” focused on molecular mechanisms of gene regulation, particularly transcriptional control in SPEM development. Cluster 2 (green) centered on cellular plasticity, featuring key terms like “stem-cells,” “metaplasia,” and “chief cells,” highlighting stem cell-driven reprogramming in gastric mucosa. Cluster 3 (blue) emphasized clinical pathology, with “intestinal metaplasia,” “carcinogenesis,” and “inflammation” underscoring the progression from *H. pylori* infection to premalignant lesions. Cluster 4 (brown–green) captured tissue-specific dynamics, including “stomach” and “differentiation,” while Cluster 5 (purple), anchored by “cancer” and “gastric metaplasia,” delineated the transition from metaplasia to malignancy. This clustering reveals the field’s multidisciplinary approach, spanning molecular, cellular, and clinical investigations of SPEM pathways.

The CiteSpace-derived keyword time-zone map and Burst Keywords visualized the temporal evolution of research on signaling pathways associated with SPEM pathogenesis from 2004 to 2025 (**Figures 6C**, **D**). Early-phase clusters (2004–2010) were dominated by high-frequency terms such as “Helicobacter pylori”, “NF-κB”, and “gastric adenocarcinoma”, underscoring the initial focus on infection-triggered inflammatory pathways that initiate SPEM. Between 2011 and 2016, the emergence of “Wnt/β-catenin”, “Notch”, and “TFF2” signaled a mechanistic shift toward delineating the intracellular networks governing chief-cell transdifferentiation. Since 2017, a rapid expansion of terms including “single-cell RNA-seq”, “chief-cell plasticity”, “organoids”, “Lgr5+ stem cells”, and “YAP” has formed densely interconnected clusters, indicating that technological advances are now dissecting microenvironmental signaling crosstalk and cellular heterogeneity within SPEM lesions.

### 3.6 Co-cited references and reference explosion

Our bibliometric analysis using VOSviewer and CiteSpace delineates the intellectual foundations and evolving trends in signaling pathways in SPEM pathogenesis. Co-citation network analysis identified highly cited references (≥10 citations) that have shaped the field, with seminal contributions including Nam et al. (2010), whose discovery of chief cells as SPEM progenitors (16 citations) established a cellular basis for metaplastic transformation, and Goldenring et al. (2010), whose conceptual framework redefined SPEM-intestinal metaplasia relationships (12 citations) (**Table 7**).

**Table 7:** Top 10 co-cited articles in the field of on signaling pathways in SPEM pathogenesis.

Rank	Author/year	Title	Journal	Citations
1	NAM KT, 2010	Mature chief cells are cryptic progenitors for metaplasia in the stomach	GASTROENTEROLOGY	16
2	NOMURA S, 2004	Spasmolytic polypeptide expressing metaplasia to preneoplasia in H. felis-infected mice	GASTROENTEROLOGY	16
3	SCHMIDT PH, 1999	Identification of a metaplastic cell lineage associated with human gastric adenocarcinoma	LABORATORY INVESTIGATION	16
4	LEFEBVRE O, 1996	Gastric mucosa abnormalities and tumorigenesis in mice lacking the pS2 trefoil protein	SCIENCE	13
5	GOLDENRING JR, 2010	Spasmolytic polypeptide-expressing metaplasia and intestinal metaplasia: time for reevaluation of metaplasias and the origins of gastric cancer	GASTROENTEROLOGY	12
6	NOZAKI K, 2008	A molecular signature of gastric metaplasia arising in response to acute parietal cell loss	GASTROENTEROLOGY	12
7	WEIS VG, 2013	Heterogeneity in mouse spasmolytic polypeptide-expressing metaplasia lineages identifies markers of metaplastic progression	GUT	12
8	CORREA P, 1992	Human gastric carcinogenesis: a multistep and multifactorial process–First American Cancer Society Award Lecture on Cancer Epidemiology and Prevention	CANCER RESEARCH	11
9	WADA T, 2013	Functional role of CD44v-xCT system in the development of spasmolytic polypeptide-expressing metaplasia	CANCER SCIENCE	11
10	HUH WJ, 2012	Tamoxifen induces rapid, reversible atrophy, and metaplasia in mouse stomach	GASTROENTEROLOGY	10

**Figure 7:**
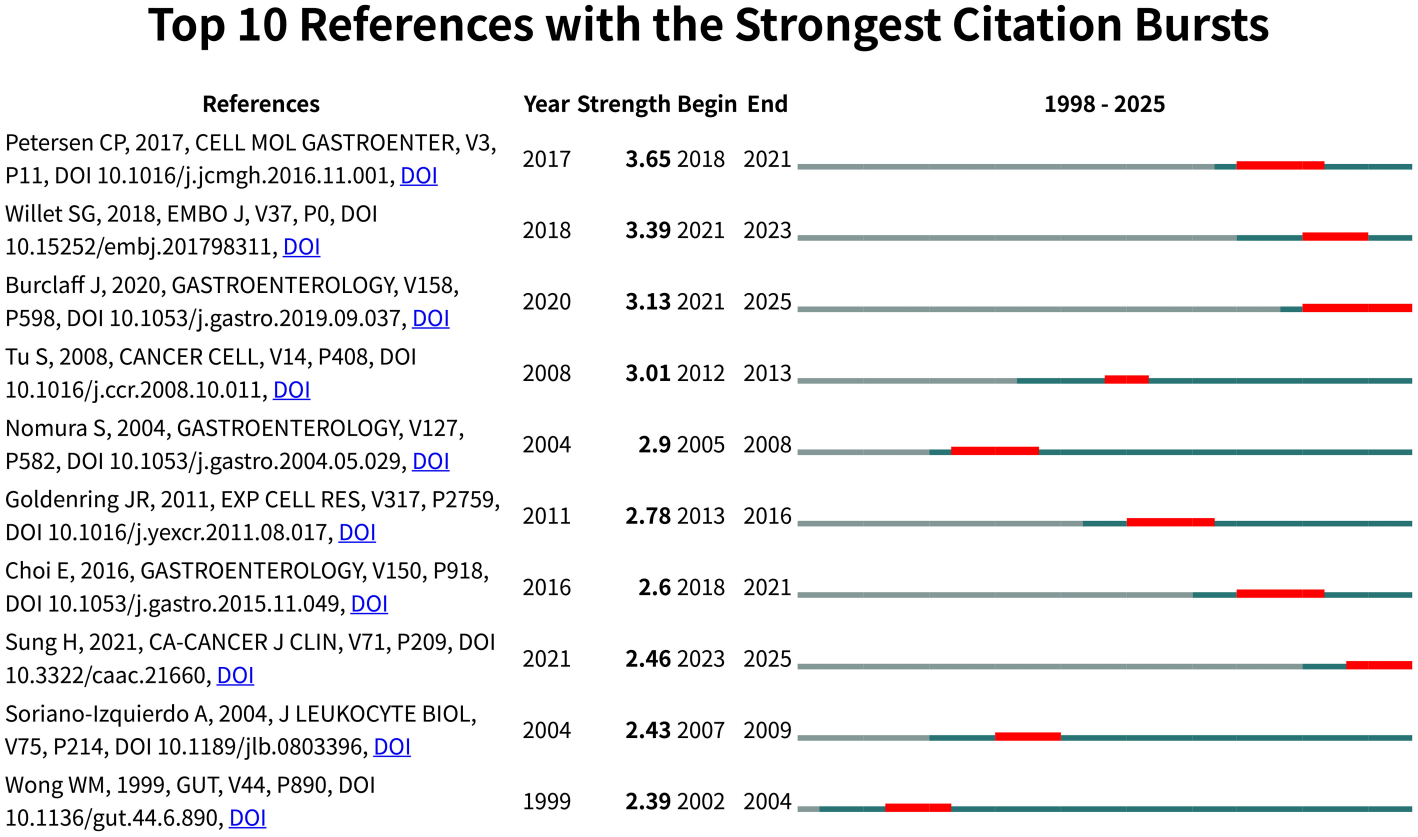
Visual analysis of reference bursts. Note: Intensity reflects the frequency of citations; red color indicates frequency of citations; green bars indicate fewer citations.

Citation burst detection (**Figure 7**) revealed Petersen et al. (2017) as the strongest burst (strength=3.65, 2018-2021), highlighting Wnt/β-catenin signaling in SPEM plasticity, while three contemporary studies - Willet et al. (2018) on Notch signaling, Burclaff et al. (2020) employing single-cell RNA sequencing, and Sung et al. (2021) focusing on clinical translation - demonstrate sustained citation surges projected through 2025. Notably, 70% of burst articles emerged post-2016, clustering around two cutting-edge themes: growth factor signaling in mucosal repair (4/10) and molecular regulation of stem cell fate (3/10).

Temporal analysis uncovered a paradigm shift from early histopathological characterization (1992-2004) to contemporary mechanistic dissection (2016-present) in SPEM pathogenesis, with Gastroenterology serving as the predominant journal (5/10 top-cited articles). This progression from phenotypic observation to pathway-level understanding reflects the maturation of SPEM research into an integrated “phenotype-signaling-translation” framework, offering novel insights into gastric carcinogenesis.

## 4 Discussion

Based on the literature retrieved from WoSCC, the ongoing debate regarding the cellular origins of spasmolytic polypeptide-expressing metaplasia (SPEM) reveals a nuanced landscape. Bibliometric analysis of the available studies indicates near-equivalent research attention to both chief cell transdifferentiation and stem cell origin theories, with 12 publications supporting the former and 11 supporting the latter. The chief cell transdifferentiation hypothesis, supported by a substantial body of research, demonstrates that mature chief cells can undergo reprogramming through multiple molecular mechanisms in response to gastric mucosal injury. These mechanisms include Stratifin (SFN)-mediated EGFR/ERK signaling activation (52), TERT/Wnt/β-catenin pathway activation driven by *H. pylori* infection (21), and metabolic reprogramming through paligenosis accompanied by large-scale organelle clearance termed “cathartocytosis” (19, 53). Conversely, the stem cell origin theory has gained significant momentum in recent investigations. A pivotal 2025 study employing advanced lineage tracing techniques demonstrated that Tff2+ corpus isthmus progenitor cells serve as the primary source of SPEM formation, with the capacity to progress directly to dysplasia following oncogenic mutations such as Kras, bypassing the metaplastic stage (54). Notably, chronic inflammatory microenvironment plays a crucial role in SPEM development. Research shows that IL33 can promote metaplasia formation in gastritis-prone mice by triggering eosinophil-dependent events, providing important mechanistic insights into how inflammatory microenvironment influences SPEM origins (55). Temporal analysis of publication trends reveals that although the chief cell transdifferentiation theory dominated earlier research, the stem cell origin hypothesis has increasingly gained traction in recent studies, particularly through the application of advanced lineage tracing and single-cell sequencing technologies (54). This evolutionary trend in research focus underscores the necessity for developing more specific cell-targeting techniques to ultimately resolve this fundamental controversy.

Based on our comprehensive bibliometric analysis, we have systematically delineated the key signaling pathways implicated in spasmolytic polypeptide-expressing metaplasia (SPEM) pathogenesis (**Supplementary Table 2**). This synthesis integrates evidence from multiple experimental models and human studies, identifying conserved pathway modules—including IL-17RC/NF-κB-mediated inflammation (56), YAP-driven proliferation (57), ROS-NRF2 oxidative stress response (58), and Notch signaling dysregulation (59, 60)—that operate across various gastric injury contexts. These pathways collectively drive characteristic pathological processes: oxyntic atrophy through parietal cell loss, chief cell transdifferentiation, and subsequent mucous cell metaplasia.

The resulting molecular framework elucidates the mechanistic basis of SPEM development and progression, providing a foundation for clinically relevant applications. These include the identification of potential tissue and circulating biomarkers for SPEM detection and risk stratification, as well as the development of targeted interventions to halt metaplastic progression in high-risk patients. This pathway-oriented perspective advances our understanding of gastric carcinogenesis and supports the development of precision medicine approaches for managing gastric preneoplastic conditions.

### 4.1 General information

This study employs bibliometric analysis combined with visualization techniques to preliminarily examine the current landscape of signaling pathways in SPEM pathogenesis. The evolution of research focus from an initial phase of descriptive pathology (1998–2011), which primarily established the clinical correlates of SPEM, to a current era of mechanistic inquiry (2012–present) aligns with broader trends in biomedical science. This shift has been propelled by the widespread adoption of single-cell technologies and multi-omics approaches, which enable the deconvolution of cellular heterogeneity, the construction of high-resolution spatiotemporal maps of the gastric mucosa, and the systematic elucidation of the molecular mechanisms driving SPEM pathogenesis (21, 52, 61). The observed publication growth (peak of 8 in 2023) coupled with a relatively modest H-index (6) suggests an expanding but still maturing field, where increased research activity has yet to produce proportionally high-impact breakthroughs. This pattern may reflect the inherent complexity of SPEM pathophysiology and the technical challenges in studying gastric mucosal reprogramming.

Journal analysis reveals Gastroenterology’s central role (10.11% of publications), underscoring its position as the primary forum for SPEM research. However, the limited presence in high-impact clinical journals points to a critical translational gap. This disparity likely stems from both the fundamental nature of current SPEM investigations and the challenges in connecting molecular mechanisms to clinical applications.

Geographic distribution shows concentrated expertise in the U.S., China, and Japan - nations with well-established gastroenterology research ecosystems. While these countries serve as important bridges in collaboration networks (centrality ≥0.10), the overall sparse connectivity (density=0.1107) suggests untapped potential for international synergy. The emergence of specialized author clusters, particularly around Goldenring JR and Mills JC’s work on chief cell plasticity, has been instrumental in defining current paradigms. However, the field would benefit from broader participation to foster innovation and diversify research perspectives.

These findings carry important implications for future research directions. First, the development of structured international consortia could accelerate progress by combining complementary expertise. Second, targeted efforts to bridge basic and clinical research could unlock therapeutic potential. Finally, strategic integration with related fields, particularly cancer stem cell biology and mucosal immunology, may yield novel insights into SPEM pathogenesis.

### 4.2 Knowledge base

The co-citation network analysis has delineated the foundational knowledge structure of SPEM research, revealing two pivotal intellectual pillars. The first pillar comprises seminal works establishing the cellular basis of SPEM, most notably Nam et al.’s identification of chief cells as metaplastic progenitors (62) (16 citations) and Goldenring et al.’s conceptual framework for SPEM-intestinal metaplasia transitions (16) (12 citations). These studies provided the histological and conceptual foundation for understanding metaplastic transformation in gastric mucosa.

The second pillar consists of mechanistic studies that have driven recent advances, as evidenced by citation bursts. The strongest burst (Petersen et al. (63); strength=3.65) reflects the field’s focus on Wnt/β-catenin signaling in SPEM plasticity, He et al (21). elucidated that the CagA protein from *H. pylori* stabilizes TERT protein by inhibiting its SYVN1-mediated ubiquitination, leading to TERT accumulation in chief cells. The accumulated TERT then functions as a co-activator for the β-catenin transcriptional complex, resulting in the hyperactivation of the Wnt/β-catenin signaling pathway. This sustained activation directly promotes the dedifferentiation of mature chief cells into SPEM cells, establishing a direct molecular link between bacterial infection and metaplasia initiation. while contemporary bursts (Willet et al. (64); Burclaff et al. (65); Sung et al. (66)) highlight three critical research fronts: the molecular circuitry of paligenosis, wherein a transient state of mTORC1 inhibition and autophagic-lysosomal activation enables the de-differentiation of mature cells into a proliferative metaplastic state; the cellular origin of pre-neoplasia, with lineage-tracing evidence establishing post-mitotic chief cells as the primary source of SPEM, fundamentally reshaping models of gastric carcinogenesis; and the pathogenic transition from reparative to pre-malignant metaplasia, driven by persistent injury signals that sustain mTORC1-driven proliferation and suppress cellular re-differentiation pathways. Notably, 70% of high-burst publications emerged post-2016, clustered around mucosal repair mechanisms (40%) and stem cell fate determination (30%), indicating the field’s current trajectory toward molecular-level understanding.

This knowledge evolution follows a clear temporal pattern, transitioning from early histopathological characterization (1992-2004) to contemporary pathway dissection (2016-present), with Gastroenterology serving as the primary dissemination venue (50% of top-cited articles). While this progression from histopathology to molecular dissection has advanced the field, resolution of cellular origin controversies remains critical for fully understanding SPEM’s role in gastric cancer.

### 4.3 Hotspot evolution, knowledge structure and emerging topics

Through systematic analysis of keyword co-occurrence and temporal evolution patterns, our study delineates the dynamic landscape of global research into the signaling pathways underlying SPEM pathogenesis. The keyword density map reveals three predominant research axes that have shaped the field’s development: gastric carcinogenesis (represented by high-frequency terms “cancer” and “metaplasia”), cellular reprogramming mechanisms (“stem-cells” and “differentiation”), and infection-driven pathogenesis (“Helicobacter-pylori”). These core themes are interconnected through pivotal conceptual bridges, particularly gene expression regulation (centrality=0.61) and *H. pylori* interactions (centrality=0.4), which serve as critical nodes linking molecular mechanisms with pathological outcomes.

The co-occurrence network analysis resolved these research themes into five distinct yet interconnected clusters, demonstrating the field’s multidisciplinary nature. Cluster-level examination shows a clear progression from fundamental molecular investigations (gene regulation and transcriptional control) through cellular plasticity studies to clinical pathology applications.

The structural characteristics of SPEM research indicate that this field is currently in a transitional phase, evolving from phenomenological descriptions toward an integrated understanding of gastric mucosal transformation signaling networks, while still lacking a mature theoretical framework. Persistent controversies regarding cellular origins (isthmus stem cells vs. chief cell transdifferentiation) and key driving pathways (22) highlight fundamental challenges in mechanistic elucidation. Our analysis reveals that with the application of novel technologies like single-cell sequencing, recent research trends have gradually shifted toward favoring the stem cell theory (67), although the transdifferentiation hypothesis maintains its explanatory power in specific experimental models. This theoretical divergence is reflected in our co-citation network analysis, demonstrating the formation of distinct research clusters around stem cell-related pathways (e.g., Wnt/Notch) versus transdifferentiation-associated signals (e.g., mTOR). However, our theory-neutral approach also uncovered nascent efforts to bridge this divide, suggesting that the two mechanisms may not be mutually exclusive. To objectively present this complex landscape, we employed a theory-neutral term extraction approach combined with temporal evolution analysis and multidimensional visualization techniques, striving to comprehensively capture the research dynamics in this field.

Temporal mapping of keyword emergence reveals three characteristic phases in the field’s evolution. The early phase (2004-2010) focused predominantly on infection-induced inflammatory pathways, particularly *H. pylori*-mediated NF-κB activation (68). A transitional phase (2011-2017) witnessed growing emphasis on intracellular signaling cascades, including Wnt/β-catenin (21) and Notch pathways (60). Most recently (2018-2025), the field has embraced advanced single-cell technologies and microenvironmental analyses, as evidenced by the emergence of terms like “single-cell RNA-seq”, “organoids”, and “YAP signaling”. Building on this technological evolution, a paradigm shift is now underway in understanding SPEM pathogenesis, moving from a reductionist focus on linear pathways to an integrative appreciation of spatiotemporal network dynamics. This evolution in research focus, as revealed by the keyword mapping, has crystallized into a new paradigm: the understanding of SPEM pathogenesis has now shifted from a linear pathway model to a dynamic spatiotemporal network paradigm. Reflecting the technologies emblematic of the most recent phase, integrated data from single-cell RNA-seq and spatial transcriptomics are delineating a complex signaling geography within SPEM lesions. Studies consistently report a compartmentalization where the lesion core is dominated by stress-responsive pathways like the IFN-γ/STAT1 axis (69), which promotes epithelial proliferation and suppresses immune cell infiltration. In contrast, the periphery is enriched in fate-determining signals such as Wnt (21, 70) and BMP (71, 72), which repress chief cell differentiation genes and drive a progenitor-like transcriptional program. Furthermore, the use of organoid models, another hallmark of this period, has enabled temporal analysis through live imaging, defining a critical sequence of events: a transient Notch pulse activates downstream effectists like Hes1 to launch the dedifferentiation of chief cells (59), while sustained EGFR signaling through its MAPK effector pathway is required to maintain the mucinous metaplastic phenotype (73). This refined spatiotemporal framework, a direct product of the field’s technological maturation, underscores the inability of earlier, static models to explain the disease process and provides a mechanistic rationale for therapeutic strategies that target specific signaling nodes based on their spatial context and temporal function.

The evolving hotspot patterns demonstrate how technological advancements have progressively enabled deeper investigation of SPEM pathogenesis, from initial histopathological descriptions to current single-cell level analyses. This trajectory underscores the field’s ongoing transformation into a sophisticated, multidimensional research domain that integrates molecular, cellular, and microenvironmental perspectives on gastric mucosal transformation. Future research directions should aim to strengthen connections between these established knowledge domains while addressing the identified translational opportunities.

### 4.4 Signaling pathways in SPEM pathogenesis: an integrated analysis

SPEM pathogenesis initiates through coordinated dysregulation of inflammatory signaling pathways that drive chief cell dedifferentiation. Sustained exposure to inflammatory stimuli like H. pylori infection activates NF-κB and STAT3 signaling in mature chief cells (74), leading to epigenetic silencing of master differentiation transcription factors including MIST1 and PGC (59, 67). This reprogramming is amplified through oxidative stress pathways, where reactive oxygen species activate the NRF2-HO-1 axis (58), further promoting the expression of metaplastic markers such as TFF2 and CD44v9. Single-cell transcriptomic analyses reveal that IL-11/STAT3 signaling (75) specifically orchestrates the transition from quiescent chief cells to proliferative SPEM precursors through sequential suppression of mature zymogenic gene programs and activation of progenitor-like transcriptional networks.

The transition to established SPEM involves convergent activation of developmental signaling pathways that lock cells in a metaplastic state. Wnt/β-catenin signaling emerges as a central regulator, with recent studies demonstrating that H. pylori CagA protein stabilizes TERT to potentiate β-catenin transcriptional activity (21). Simultaneously, YAP/TAZ signaling responds to biomechanical cues from the stiffening microenvironment, driving proliferation while suppressing apoptosis through regulation of anti-apoptotic proteins (57). This mechanosensitive signaling interacts with Wnt pathway components to create a self-reinforcing circuit that maintains dedifferentiation. Complementing these pathways, BMP signaling (72) - normally responsible for promoting chief cell maturation - becomes suppressed, removing critical differentiation pressure and permitting metaplastic persistence.

At the transcriptional level, SPEM cells exhibit a distinct enhancer landscape that reinforces the metaplastic program. Epigenomic profiling reveals super-enhancer reorganization around key transcription factor genes including SOX9 and CDX2 (56), which collaborate to maintain the SPEM transcriptional identity. These enhancer alterations are mediated through the coordinated action of inflammatory and developmental signaling pathways, with STAT3 directly binding to and remodeling enhancer regions controlling SPEM-associated genes (75). The resulting epigenetic state creates a stable transcriptional memory that persists even after removal of initial inflammatory triggers, explaining the irreversible nature of established SPEM lesions.

Metabolic reprogramming and microenvironmental crosstalk constitute the third pillar of SPEM maintenance. mTORC1 activation coordinates autophagic flux and lipid metabolism to support the bioenergetic demands of rapidly dividing SPEM cells (76). This metabolic adaptation interfaces with inflammatory signaling through mitochondrial ROS production, which further sustains NF-κB activation (58). The metaplastic epithelium actively remodels its microenvironment through secretion of IL-33 and other alarmins that recruit and polarize type 2 innate lymphoid cells and M2 macrophages, creating a pro-metaplastic inflammatory niche (58). Additionally, mesenchymal cells respond through aberrant PKA signaling (77), secreting factors that promote epithelial hyperplasia. This intricate network of epithelial-stromal-immune interactions creates a resilient signaling ecosystem that sustains SPEM and presents challenges for therapeutic intervention, necessitating multi-target approaches that address both cell-autonomous and non-autonomous signaling mechanisms.

## 5 Limitations

This study presents a systematic analysis of current signaling pathways in SPEM pathogenesis research, demonstrating several key features: it employs multidimensional scientometric indicators (citation frequency, H-index, etc.) for comprehensive evaluation and utilizes network visualization to clearly delineate international collaboration patterns. The research acknowledges certain limitations: the Web of Science-restricted sample (n=89) may not fully represent the field, potentially introducing selection bias, and citation-based metrics could overemphasize high-impact journals while undervaluing clinically relevant studies. A key limitation is the current underrepresentation of clinical translation studies in high-impact oncology and bioengineering journals, underscoring the need for stronger translational research frameworks. The observed geographical dominance (U.S./China/Japan) may reflect funding disparities rather than scientific capacity, and our institutional analysis didn’t distinguish basic/clinical research. Methodologically, keyword-based searches risk terminology omissions, and we lacked cross-database validation (e.g., Scopus comparisons) or Delphi consensus due to the study’s retrospective design—though these represent important avenues for future work alongside systematic quality appraisal and alternative metrics.

## 6 Conclusion and future perspectives

This comprehensive bibliometric analysis delineates the global research landscape and evolving trends in signaling pathways associated with spasmolytic polypeptide-expressing metaplasia pathogenesis. The findings reveal a dynamically growing field since 1998, characterized by substantial contributions from leading gastroenterology research institutions in the United States, China, and Japan. The intellectual architecture of this domain demonstrates a progressive evolution from foundational histopathological characterization to contemporary investigations focusing on dysregulated signaling networks, particularly inflammatory pathways involving IL-33 signaling, developmental pathways including Wnt and β-catenin signaling, and metabolic pathways centered around mTORC1 regulation. The analysis reveals several persistent challenges requiring strategic attention. A significant translational gap remains between mechanistic discoveries and their clinical applications, while limited international collaboration and insufficient interdisciplinary integration continue to hinder the field’s development. Our analysis delineates that the SPEM field’s knowledge structure is primarily experimental. The relatively isolated co-citation network position of the computational biology paradigm, even with the advent of techniques like single-cell RNA sequencing (21, 78, 79), indicates that a truly cross-disciplinary integration remains an imperative for the field.

Future research directions should prioritize several strategic areas. First, resolving fundamental biological questions through integrated multi-omics methodologies represents an urgent priority. Advanced techniques combining single-cell RNA sequencing with spatial transcriptomics offer unprecedented capability to delineate the developmental trajectory of MUC6 and TFF2-expressing SPEM cells within their native gastric microenvironment (80, 81). Second, validating conserved pathway modules as therapeutic targets requires innovative model systems and experimental approaches. Gastric organoids have emerged as particularly valuable tools for this purpose, as demonstrated by recent work utilizing organoids derived from transgenic mouse models (82). These systems enable precise investigation of signaling pathways involved in epithelial damage repair through sophisticated techniques including single-cell photodamage and real-time calcium imaging. Studies employing pharmacological modulators of epidermal growth factor receptor and chemokine receptors in gastric organoids have successfully identified critical signaling requirements for epithelial repair processes. This organoid-based approach provides a powerful platform for validating therapeutic targets within key signaling axes while maintaining physiological relevance, making it particularly suitable for investigating pathway modules conserved in gastric precancerous conditions. The integration of artificial intelligence and machine learning is revolutionizing the investigation of signaling pathways in SPEM pathogenesis. These computational approaches enable comprehensive analysis of multi-omics data, providing novel insights into SPEM regulatory networks. Advanced algorithms can reconstruct dynamic signaling networks by integrating single-cell and spatial transcriptomic data, identifying critical hub molecules such as SOX9 within the SPEM microenvironment (83).

Establishing robust international collaborative networks following successful consortium models represents another critical success factor, promoting equitable resource sharing and standardized methodology implementation. By strategically embracing these priorities, particularly through computational and biological integration, the research community can accelerate the development of clinically relevant biomarkers and precision therapeutics for gastric precancerous lesions. This bibliometric analysis thus provides both a comprehensive reference of field development and a strategic roadmap for future research directions aimed at improving gastric cancer prevention outcomes.

## Data Availability

The raw data supporting the conclusions of this article will be made available by the authors, without undue reservation.

## References

[1] Bray F Laversanne M Sung H Ferlay J Siegel RL Soerjomataram I . Global cancer statistics 2022: GLOBOCAN estimates of incidence and mortality worldwide for 36 cancers in 185 countries. CA Cancer J Clin. (2024) 74:229–63. doi: 10.3322/caac.21834, PMID: 38572751

[2] Zheng RS Chen R Han BF Wang SM Li L Sun KX . Cancer incidence and mortality in China, 2022. Zhonghua Zhong Liu Za Zhi. (2024) 46:221–31. doi: 10.3760/cma.j.cn112152-20240119-00035, PMID: 38468501

[3] Humans IWGotEoCRt . Biological agents. IARC Monogr Eval Carcinog Risks Hum. (2012) 100:1–441. PMC4781184 23189750

[4] Malfertheiner P Camargo MC El-Omar E Liou J-M Peek R Schulz C . Helicobacter pylori infection. Nat Rev Dis Primers. (2023) 9:19. doi: 10.1038/s41572-023-00431-8, PMID: 37081005 PMC11558793

[5] Wizenty J Sigal M . Gastric stem cell biology and helicobacter pylori infection. In: Backert S , editor. Helicobacter pylori and Gastric Cancer. Springer Nature Switzerland, Cham (2023). p. 1–24. 10.1007/978-3-031-47331-9_1 38231213

[6] Portillo-Miño JD Calderón JJ Ruiz-García E Monge C . Myeloid-derived suppressor cells modulation in the context of tumor microenvironment for gastric cancer. Clin Trans Oncol. (2025) 27:4342–58. doi: 10.1007/s12094-025-03960-8, PMID: 40555972

[7] Holokai L Chakrabarti J Broda T Chang J Hawkins JA Sundaram N . Increased programmed death-ligand 1 is an early epithelial cell response to helicobacter pylori infection. PloS Pathog. (2019) 15:e1007468. doi: 10.1371/journal.ppat.1007468, PMID: 30703170 PMC6380601

[8] Park JY Georges D Alberts CJ Bray F Clifford G Baussano I . Global lifetime estimates of expected and preventable gastric cancers across 185 countries. Nat Med. (2025) 31:3020–7. doi: 10.1038/s41591-025-03793-6, PMID: 40624406 PMC12443596

[9] Correa P . Human gastric carcinogenesis: a multistep and multifactorial process–First American Cancer Society Award Lecture on Cancer Epidemiology and Prevention. Cancer Res. (1992) 52:6735–40., PMID: 1458460

[10] Sugano K Moss SF Kuipers EJ . Gastric intestinal metaplasia: real culprit or innocent bystander as a precancerous condition for gastric cancer? Gastroenterology. (2023) 165:1352–66.e1. doi: 10.1053/j.gastro.2023.08.028, PMID: 37652306

[11] Ye Q Zhu Y Ma Y Wang Z Xu G . Emerging role of spasmolytic polypeptide-expressing metaplasia in gastric cancer. J Gastrointest Oncol. (2024) 15:2673–83. doi: 10.21037/jgo-24-508, PMID: 39816029 PMC11732338

[12] Zavros Y Merchant JL . The immune microenvironment in gastric adenocarcinoma. Nat Rev Gastroenterol Hepatol. (2022) 19:451–67. doi: 10.1038/s41575-022-00591-0, PMID: 35288702 PMC9809534

[13] Lenti MV Rugge M Lahner E Miceli E Toh BH Genta RM . Autoimmune gastritis. Nat Rev Dis Primers. (2020) 6:57. doi: 10.1038/s41572-020-0198-5, PMID: 32647173

[14] Rugge M Sugano K Sacchi D Sbaraglia M Malfertheiner P . Gastritis: an update in 2020. Curr Treat Options Gastroenterol. (2020) 18:488–503. doi: 10.1007/s11938-020-00298-8

[15] Hong X Li H Lin Y Luo L Xu W Kang J . Efficacy and potential therapeutic mechanism of Weiwei decoction on Spasmolytic polypeptide-expressing metaplasia in Helicobacter pylori-infected and Atp4a-knockout mice. J Ethnopharmacol. (2024) 319:117062. doi: 10.1016/j.jep.2023.117062 (1872-7573 (Electronic))., PMID: 37598768

[16] Goldenring JR Mills JC . Cellular plasticity, reprogramming, and regeneration: metaplasia in the stomach and beyond. Gastroenterology. (2022) 162:415–30. doi: 10.1053/j.gastro.2021.10.036, PMID: 34728185 PMC8792220

[17] Caldwell B Meyer AR Weis JA Engevik AC Choi E . Chief cell plasticity is the origin of metaplasia following acute injury in the stomach mucosa. Gut. (2022) 71:1068–77. doi: 10.1136/gutjnl-2021-325310, PMID: 34497145 PMC8901801

[18] Zhao Y Deng Z Ma Z Zhang M Wang H Tuo B . Expression alteration and dysfunction of ion channels/transporters in the parietal cells induces gastric diffused mucosal injury. BioMed Pharmacother. (2022) 148:112660. doi: 10.1016/j.biopha.2022.112660, PMID: 35276516

[19] Miao ZF Sun JX Huang XZ Bai S Pang MJ Li JY . Metaplastic regeneration in the mouse stomach requires a reactive oxygen species pathway. Dev Cell. (2024) 59:1175–91.e7. doi: 10.1016/j.devcel.2024.03.002, PMID: 38521055

[20] Meyer AR Goldenring JR . Injury, repair, inflammation and metaplasia in the stomach. J Physiol. (2018) 596:3861–67. doi: 10.1113/jp275512, PMID: 29427515 PMC6117566

[21] He LJ Zhang X Zhang SW Wang Y Hu WC Li J . H. Pylori-facilitated TERT/wnt/β-catenin triggers spasmolytic polypeptide-expressing metaplasia and oxyntic atrophy. Adv Sci. (2024) 12:16. doi: 10.1002/advs.202401227, PMID: 39587848 PMC11744579

[22] Kinoshita H Hayakawa Y Koike K . Metaplasia in the stomach-precursor of gastric cancer? Int J Mol Sci. (2017) 18:2063. doi: 10.3390/ijms18102063, PMID: 28953255 PMC5666745

[23] Aihara E Engevik KA Montrose MH . Trefoil factor peptides and gastrointestinal function. Annu Rev Physiol. (2017) 79:357–80. doi: 10.1146/annurev-physiol-021115-105447, PMID: 27992733 PMC5474939

[24] Chong Y Yu D Lu Z Nie F . Role and research progress of spasmolytic polypeptide−expressing metaplasia in gastric cancer (Review). Int J Oncol. (2024) 64:33. doi: 10.3892/ijo.2024.5621, PMID: 38299264 PMC10836494

[25] Fan R Wu K Yang J Zhu B Jiang T Liu Y . Proteomic landscape of small extracellular vesicles derived from gastric juice and identified TFF2 as a specific biomarker. Int J Nanomed. (2025) 20:6929–48. doi: 10.2147/ijn.S516605, PMID: 40458745 PMC12129028

[26] Kuo HY Chang WL Yeh YC Tsai YC Wu CT Cheng HC . Serum Level of Trefoil Factor 2 can Predict the Extent of Gastric Spasmolytic Polypeptide-Expressing Metaplasia in the H. pylori-Infected Gastric Cancer Relatives. Helicobacter. (2017) 22. doi: 10.1111/hel.12320, PMID: 27220894

[27] Yan Z Liu Y Yuan Y . The plasticity of epithelial cells and its potential in the induced differentiation of gastric cancer. Cell Death Discov. (2024) 10:512. doi: 10.1038/s41420-024-02275-x, PMID: 39719478 PMC11668900

[28] Liu S Zhang N Ji X Yang S Zhao Z Li P . Helicobacter pylori CagA promotes gastric cancer immune escape by upregulating SQLE. Cell Death Dis. (2025) 16:17. doi: 10.1038/s41419-024-07318-w, PMID: 39809787 PMC11733131

[29] He L Zhang X Zhang S Wang Y Hu W Li J . H. Pylori-facilitated TERT/wnt/β-catenin triggers spasmolytic polypeptide-expressing metaplasia and oxyntic atrophy. Adv Sci. (2025) 12:2401227. doi: 10.1002/advs.202401227, PMID: 39587848 PMC11744579

[30] Cui X Chang M Wang Y Liu J Sun Z Sun Q . Helicobacter pylori reduces METTL14-mediated VAMP3 m(6)A modification and promotes the development of gastric cancer by regulating LC3C-mediated c-Met recycling. Cell Death Discov. (2025) 11:13. doi: 10.1038/s41420-025-02289-z, PMID: 39827141 PMC11742886

[31] Engevik KA Hanyu H Matthis AL Zhang T Frey MR Oshima Y . Trefoil factor 2 activation of CXCR4 requires calcium mobilization to drive epithelial repair in gastric organoids. J Physiol. (2019) 597:2673–90. doi: 10.1113/jp277259, PMID: 30912855 PMC6826237

[32] Son M Wang AG Keisham B Tay S . Processing stimulus dynamics by the NF-κB network in single cells. Exp Mol Med. (2023) 55:2531–40. doi: 10.1038/s12276-023-01133-7 PMC10766959 38040923

[33] Jahan R Shah A Kisling SG Macha MA Thayer S Batra SK . Odyssey of trefoil factors in cancer: Diagnostic and therapeutic implications. Biochim Biophys Acta Rev Cancer. (2020) 1873:188362. doi: 10.1016/j.bbcan.2020.188362, PMID: 32298747

[34] Nishida T Nakamatsu D Matsumoto K Yamamoto M . Has the issue of the “point of no return” in gastric carcinogenesis already been resolved? Gastrointest Endosc. (2021) 94:199. doi: 10.1016/j.gie.2021.02.006, PMID: 34148572

[35] Hoffmann W . Trefoil factor family (TFF) peptides and their links to inflammation: A re-evaluation and new medical perspectives. Int J Mol Sci. (2021) 22. doi: 10.3390/ijms22094909, PMID: 34066339 PMC8125380

[36] Xu Y Liu Y Kan X Li X Sun H Zhang J . (2023). Application research of image processing and visualization thchnology in gastric mucosal protective factors using artificial intelligence, in: 2023 International Conference on Telecommunications, Electronics and Informatics (ICTEI), 11–13 Sept. 2023. Lisbon, Portugal: IEEE.

[37] Zhang T Tang X . Chemoprevention strategies for precancerous gastric lesions beyond helicobacter pylori eradication. Qjm. (2025) 118:385–409. doi: 10.1093/qjmed/hcaf030, PMID: 39880367

[38] Ho SWT Sheng T Xing M Ooi WF Xu C Sundar R . Regulatory enhancer profiling of mesenchymal-type gastric cancer reveals subtype-specific epigenomic landscapes and targetable vulnerabilities. Gut. (2023) 72:226–41. doi: 10.1136/gutjnl-2021-326483, PMID: 35817555

[39] Yeoh KG Tan P . Mapping the genomic diaspora of gastric cancer. Nat Rev Cancer. (2022) 22:71–84. doi: 10.1038/s41568-021-00412-7, PMID: 34702982

[40] Chen C . CiteSpace II: Detecting and visualizing emerging trends and transient patterns in scientific literature. J Am Soc Inf Sci Technol. (2006) 57:359–77. doi: 10.1002/asi.20317

[41] Kirby A . Exploratory bibliometrics: using VOSviewer as a preliminary research tool. Publications. (2023) 11:10. doi: 10.3390/publications11010010

[42] Lu W Huang S Yang J Bu Y Cheng Q Huang Y . Detecting research topic trends by author-defined keyword frequency. Inf Process Manage. (2021) 58:102594. doi: 10.1016/j.ipm.2021.102594

[43] Boyack KW Klavans R . Co-citation analysis, bibliographic coupling, and direct citation: Which citation approach represents the research front most accurately? J Am Soc Inf Sci Technol. (2010) 61:2389–404. doi: 10.1002/asi.21419

[44] Kurt-Jones EA Cao L Sandor F Rogers AB Whary MT Nambiar PR . Trefoil family factor 2 is expressed in murine gastric and immune cells and controls both gastrointestinal inflammation and systemic immune responses. Infect Immun. (2007) 75:471–80. doi: 10.1128/iai.02039-05, PMID: 17101660 PMC1828407

[45] Choi E Hendley AM Bailey JM Leach SD Goldenring JR . Expression of activated ras in gastric chief cells of mice leads to the full spectrum of metaplastic lineage transitions. Gastroenterology. (2016) 150:918–+. doi: 10.1053/j.gastro.2015.11.049, PMID: 26677984 PMC4808451

[46] Dubeykovskaya Z Dubeykovskiy A Solal-Cohen J Wang TC . Secreted trefoil factor 2 activates the CXCR4 receptor in epithelial and lymphocytic cancer cell lines. J Biol Chem. (2009) 284:3650–62. doi: 10.1074/jbc.M804935200, PMID: 19064997 PMC2635042

[47] Xiong MY Chen XT Wang HM Tang X Wang QJ Li XG . Combining transcriptomics and network pharmacology to reveal the mechanism of Zuojin capsule improving spasmolytic polypeptide-expressing metaplasia. J Ethnopharmacol. (2024) 318:12. doi: 10.1016/j.jep.2023.117075, PMID: 37625606

[48] Tao DY Guan BX Li ZX Jiao M Zhou CJ Li H . Correlation of Claudin18.2 expression with clinicopathological characteristics and prognosis in gastric cancer. Pathol Res Pract. (2023) 248:12. doi: 10.1016/j.prp.2023.154699, PMID: 37487317

[49] Suzuki K Sentani K Tanaka H Yano T Suzuki K Oshima M . Deficiency of stomach-type claudin-18 in mice induces gastric tumor formation independent of H pylori infection. Cell Mol Gastroenterol Hepatol. (2019) 8:119–42. doi: 10.1016/j.jcmgh.2019.03.003, PMID: 30910700 PMC6554658

[50] Hanisch FG Bonar D Schloerer N Schroten H . Human Trefoil Factor 2 Is a Lectin That Binds α-GlcNAc-capped Mucin Glycans with Antibiotic Activity against Helicobacter pylori. J Biol Chem. (2014) 289:27363–75. doi: 10.1074/jbc.M114.597757, PMID: 25124036 PMC4183777

[51] Loncar MB Al-azzeh E Sommer PSM Marinovic M Schmehl K Kruschewski M . Tumour necrosis factor α and nuclear factor κB inhibit transcription of human TFF3 encoding a gastrointestinal healing peptide. Gut. (2003) 52:1297–303. doi: 10.1136/gut.52.9.1297, PMID: 12912861 PMC1773791

[52] Won Y Sohn Y Lee S-H Goldstein A Gangula R Mallal S . Stratifin is necessary for spasmolytic polypeptide-expressing metaplasia development after acute gastric injury. Cell Mol Gastroenterol Hepatol. (2025) 19:101521. doi: 10.1016/j.jcmgh.2025.101521, PMID: 40280276 PMC12169795

[53] Brown JW Lin X Nicolazzi GA Liu X Nguyen T Radyk MD . Cathartocytosis: Jettisoning of cellular material during reprogramming of differentiated cells. Cell Rep. (2025) 44:116070. doi: 10.1016/j.celrep.2025.116070, PMID: 40742812 PMC12478994

[54] Tu R Zheng H Zheng B Zhong Q Qian J Wu F . Tff2 marks gastric corpus progenitors that give rise to pyloric metaplasia/SPEM following injury. bioRxiv. (2025). doi: 10.1101/2025.04.09.647847 (2692-8205 (Electronic))., PMID: 40291734 PMC12027342

[55] De Salvo C Pastorelli L Petersen CP Buttò LF Buela KA Omenetti S . Interleukin 33 triggers early eosinophil-dependent events leading to metaplasia in a chronic model of gastritis-prone mice. Gastroenterology. (2021) 160:302–+. doi: 10.1053/j.gastro.2020.09.040, PMID: 33010253 PMC7755675

[56] Lang YF Han XR Liu X Ning J Hao XY Zhang HJ . Cmtm4 deficiency inhibits helicobacter pylori-induced gastric carcinogenesis. Pathol Int. (2025) 13:278–90. doi: 10.1111/pin.70020, PMID: 40432275

[57] Loe AKH Rao-Bhatia A Wei Z Kim JE Guan BX Qin Y . YAP targetome reveals activation of SPEM in gastric pre-neoplastic progression and regeneration. Cell Rep. (2023) 42:26. doi: 10.1016/j.celrep.2023.113497, PMID: 38041813

[58] Zeng X Yang MH Ye TB Feng JM Xu XH Yang HA . Mitochondrial GRIM-19 loss in parietal cells promotes spasmolytic polypeptide-expressing metaplasia through NLR family pyrin domain-containing 3 (NLRP3)-mediated IL-33 activation via a reactive oxygen species (ROS)-NRF2-Heme oxygenase-1(HO-1)-NF-κB axis. Free Radic Biol Med. (2023) 202:46–61. doi: 10.1016/j.freeradbiomed.2023.03.024, PMID: 36990300

[59] Chung WC Zhou YY Atfi A Xu KL . Downregulation of notch signaling in kras-induced gastric metaplasia. Neoplasia. (2019) 21:810–21. doi: 10.1016/j.neo.2019.06.003, PMID: 31276933 PMC6611983

[60] Demitrack ES Gifford GB Keeley TM Horita N Todisco A Turgeon DK . NOTCH1 and NOTCH2 regulate epithelial cell proliferation in mouse and human gastric corpus. Am J Physiol-Gastroint Liver Physiol. (2017) 312:G133–G44. doi: 10.1152/ajpgi.00325.2016, PMID: 27932500 PMC5338607

[61] Wang J Feng J Chen X Weng Y Wang T Wei J . Integrated multi-omics analysis and machine learning identify hub genes and potential mechanisms of resistance to immunotherapy in gastric cancer. Aging (Albany NY). (2024) 16:7331–56. doi: 10.18632/aging.205760, PMID: 38656888 PMC11087130

[62] Nam KT O’Neal R Lee YS Lee YC Coffey RJ Goldenring JR . Gastric tumor development in Smad3-deficient mice initiates from forestomach/glandular transition zone along the lesser curvature. Lab Invest. (2012) 92:883–95. doi: 10.1038/labinvest.2012.47, PMID: 22411066 PMC3584162

[63] Petersen CP Mills JC Goldenring JR . Murine models of gastric corpus preneoplasia. Cell Mol Gastroenterol Hepatol. (2016) 3:11–26. doi: 10.1016/j.jcmgh.2016.11.001 (2352-345X (Print))., PMID: 28174755 PMC5247421

[64] Willet SG Lewis MA Miao ZF Liu D Radyk MD Cunningham RL . Regenerative proliferation of differentiated cells by mTORC1-dependent paligenosis. EMBO J. (2018) 37:e98311. doi: 10.15252/embj.201798311, PMID: 29467218 PMC5881627

[65] Burclaff J Willet SG Sáenz JB Mills JC . Proliferation and differentiation of gastric mucous neck and chief cells during homeostasis and injury-induced metaplasia. Gastroenterology. (2020) 158:598–609.e5. doi: 10.1053/j.gastro.2019.09.037, PMID: 31589873 PMC7010566

[66] Sung H Ferlay J Siegel RL Laversanne M Soerjomataram I Jemal A . Global cancer statistics 2020: GLOBOCAN estimates of incidence and mortality worldwide for 36 cancers in 185 countries. CA Cancer J Clin. (2021) 71:209–49. doi: 10.3322/caac.21660, PMID: 33538338

[67] Zhang M Hu S Min M Ni Y Lu Z Sun X . Dissecting transcriptional heterogeneity in primary gastric adenocarcinoma by single cell RNA sequencing. Gut. (2021) 70:464–75. doi: 10.1136/gutjnl-2019-320368, PMID: 32532891 PMC7873416

[68] El-Zaatari M Tobias A Grabowska AM Kumari R Scotting PJ Kaye P . De-regulation of the sonic hedgehog pathway in the InsGas mouse model of gastric carcinogenesis. Br J Cancer. (2007) 96:1855–61. doi: 10.1038/sj.bjc.6603782, PMID: 17505514 PMC2359963

[69] Syu LJ El-Zaatari M Eaton KA Liu ZP Tetarbe M Keeley TM . Transgenic expression of interferon-γ in mouse stomach leads to inflammation, metaplasia, and dysplasia. Am J Pathol. (2012) 181:2114–25. doi: 10.1016/j.ajpath.2012.08.017, PMID: 23036899 PMC3509761

[70] Yu M Qin K Fan J Zhao G Zhao P Zeng W . The evolving roles of Wnt signaling in stem cell proliferation and differentiation, the development of human diseases, and therapeutic opportunities. Genes Dis. (2024) 11:101026. doi: 10.1016/j.gendis.2023.04.042, PMID: 38292186 PMC10825312

[71] Maloum F Allaire JM Gagné-Sansfaçon J Roy E Belleville K Sarret P . Epithelial BMP signaling is required for proper specification of epithelial cell lineages and gastric endocrine cells. Am J Physiol-Gastroint Liver Physiol. (2011) 300:G1065–G79. doi: 10.1152/ajpgi.00176.2010, PMID: 21415412 PMC3119118

[72] Ye W Takabayashi H Yang YT Mao M Hibdon ES Samuelson LC . Regulation of gastric lgr5+ve cell homeostasis by bone morphogenetic protein (BMP) signaling and inflammatory stimuli. Cell Mol Gastroenterol Hepatol. (2018) 5:523–38. doi: 10.1016/j.jcmgh.2018.01.007, PMID: 29930977 PMC6009760

[73] Zhang Y Yu GY Xiang Y Wu JB Jiang P Lee WH . Bm-TFF2, a toad trefoil factor, promotes cell migration, survival and wound healing. Biochem Biophys Res Commun. (2010) 398:559–64. doi: 10.1016/j.bbrc.2010.06.118, PMID: 20599756

[74] Ishii Y Shibata W Sugimori M Kaneta Y Kanno M Sato T . Activation of signal transduction and activator of transcription 3 signaling contributes to helicobacter-associated gastric epithelial proliferation and inflammation. Gastroenterol Res Pract. (2018) 2018:9. doi: 10.1155/2018/9050715, PMID: 29849601 PMC5911338

[75] Buzzelli JN O’Connor L Scurr M Chin SCN Catubig A Ng GZ . Overexpression of IL-11 promotes premalignant gastric epithelial hyperplasia in isolation from germline gp130-JAK-STAT driver mutations. Am J Physiol-Gastroint Liver Physiol. (2019) 316:G251–G62. doi: 10.1152/ajpgi.00304.2018, PMID: 30520693

[76] Miao ZF Sun JX Adkins-Threats M Pang MJ Zhao JH Wang X . DDIT4 licenses only healthy cells to proliferate during injury-induced metaplasia. Gastroenterology. (2021) 160:260–+. doi: 10.1053/j.gastro.2020.09.016, PMID: 32956680 PMC7857017

[77] Puri P Grimmett G Faraj R Gibson L Gilbreath E Yoder BK . Elevated protein kinase A activity in stomach mesenchyme disrupts mesenchymal-epithelial crosstalk and induces preneoplasis. Cell Mol Gastroenterol Hepatol. (2022) 14:643–+. doi: 10.1016/j.jcmgh.2022.06.001, PMID: 35690337 PMC9421585

[78] Busada JT Peterson KN Khadka S Xu XJ Oakley RH Cook DN . Glucocorticoids and androgens protect from gastric metaplasia by suppressing group 2 innate lymphoid cell activation. Gastroenterology. (2021) 161:637–+. doi: 10.1053/j.gastro.2021.04.075, PMID: 33971182 PMC8328958

[79] Ding L Sheriff S Sontz RA Merchant JL . Schlafen4+-MDSC in Helicobacter-induced gastric metaplasia reveals role for GTPases. Front Immunol. (2023) 14:1139391. doi: 10.3389/fimmu.2023.1139391, PMID: 37334372 PMC10272601

[80] Bockerstett KA Lewis SA Wolf KJ Noto CN Jackson NM Ford EL . Single-cell transcriptional analyses of spasmolytic polypeptide-expressing metaplasia arising from acute drug injury and chronic inflammation in the stomach. Gut. (2020) 69:1027–38. doi: 10.1136/gutjnl-2019-318930, PMID: 31481545 PMC7282188

[81] Tsubosaka A Komura D Kakiuchi M Katoh H Onoyama T Yamamoto A . Stomach encyclopedia: Combined single-cell and spatial transcriptomics reveal cell diversity and homeostatic regulation of human stomach. Cell Rep. (2023) 42:113236. doi: 10.1016/j.celrep.2023.113236, PMID: 37819756

[82] Idowu S Bertrand PP Walduck AK . Gastric organoids: Advancing the study of H. pylori pathogenesis and inflammation. Helicobacter. (2022) 27:e12891. doi: 10.1111/hel.12891, PMID: 35384141 PMC9287064

[83] Zhang G Zhang X Pan W Chen X Wan L Liu C . Dissecting the spatial and single-cell transcriptomic architecture of cancer stem cell niche driving tumor progression in gastric cancer. Adv Sci (Weinh). (2025) 12:e2413019. doi: 10.1002/advs.202413019, PMID: 39950944 PMC12079437

